# Association of Tumor-Infiltrating Lymphocytes (TILs) With Pathological Complete Response to Neoadjuvant Therapy in Breast Cancer

**DOI:** 10.7759/cureus.96076

**Published:** 2025-11-04

**Authors:** Noor Ul Ain, Sara Ishaq, Muhammad Khurrum Islam, Quratulain Badar, Kamran Chaudhry, Tooba Adil, Sudhair Abbas Bangash

**Affiliations:** 1 Oncology, King Edward Medical College, Lahore, Lahore, PAK; 2 Medical Oncology, King Edward Medical University, Mayo Hospital, Lahore, Lahore, PAK; 3 Oncology, Oncology Ward Allied Hospital Faisalabad, Lahore, PAK; 4 Department of Radiation Oncology, Dr. Ziauddin Hospital, Karachi, PAK; 5 Family Practice, Tehsil Head Quarter (THQ) Hospital, Kot Radha Kishan, Lahore, PAK; 6 Community Medicine, Jinnah Sindh Medical University, Karachi, PAK; 7 Department of Pharmacy, Sarhad University of Science and Information Technology, Peshawar, PAK

**Keywords:** breast cancer, immune response, immunotherapy, neoadjuvant therapy, oncology, pathological complete response, predictive biomarkers, treatment outcomes, tumor-infiltrating lymphocytes, tumor microenvironment

## Abstract

Background: Breast cancer remains a major global health challenge, with tumor-infiltrating lymphocytes (TILs) emerging as a potential biomarker of response to neoadjuvant therapy. This study aimed to evaluate the association between stromal TILs and pathological complete response (pCR) across different molecular subtypes of breast cancer within a South Asian population.

Materials and methods: A cross-sectional analytical study was conducted at the Department of Oncology, Sindh Medical College, Karachi, from February 15, 2025, to August 15, 2025. A total of 110 female patients with histologically confirmed breast cancer who received standard neoadjuvant therapy and subsequently underwent surgery were included. The cohort comprised all major molecular subtypes (Luminal A, Luminal B, HER2-positive, and triple-negative). HER2-positive patients received anti-HER2-targeted therapy (trastuzumab ± pertuzumab), while triple-negative patients received standard chemotherapy. Stromal TILs were evaluated on pre-treatment biopsy specimens stained with hematoxylin and eosin (H&E), following the International Immuno-Oncology Biomarker Working Group guidelines. A pre-specified cut-off of 20% was used to categorize TILs as low or high, consistent with prior studies and regional validation protocols. Statistical analyses included chi-square tests and multivariate logistic regression to identify independent predictors of pCR.

Results: The mean age of participants was 48.6 ± 10.2 years, and 58 (52.7%) were postmenopausal. Overall, 40 (36.4%) patients achieved pCR. High TILs were observed in 50 (45.5%) cases and were significantly associated with higher pCR rates compared to those with low TILs (28/50, 56.0% vs. 12/60, 20.0%; p < 0.001). In multivariate analysis, high TILs (adjusted OR 5.25; 95% CI 2.10-13.1; p < 0.001), HER2-positive subtype (adjusted OR 2.10; 95% CI 0.90-4.92; p = 0.08), and triple-negative subtype (adjusted OR 1.75; 95% CI 0.65-4.72; p = 0.27) independently predicted higher pCR rates.

Conclusion: High stromal TILs were strongly associated with improved pathological complete response following neoadjuvant therapy, particularly in HER2-positive and triple-negative breast cancer subtypes. Routine TIL assessment offers a simple, cost-effective, and clinically meaningful biomarker for predicting therapeutic response and guiding individualized treatment strategies, especially within resource-limited regional settings.

## Introduction

Breast cancer remains the most commonly diagnosed malignancy and the leading cause of cancer-related death among women worldwide [1]. According to the Global Cancer Observatory (GLOBOCAN), approximately 2.3 million new breast cancer cases and 685,000 deaths were reported in 2020, accounting for nearly one in four cancer cases and one in six cancer deaths among women globally [2,3]. In Asia, and particularly in Pakistan, breast cancer represents one of the most prevalent malignancies, with a disturbing increase in incidence among women under the age of 50 [4-6]. Despite significant advancements in screening, early detection, and multimodal therapeutic approaches, breast cancer continues to impose a major public health and socioeconomic burden.

Neoadjuvant therapy - encompassing chemotherapy, targeted therapy, and hormonal treatment administered before surgery - has become a cornerstone in breast cancer management. Its benefits include tumor downstaging, increasing eligibility for breast-conserving surgery, and providing early insight into treatment efficacy [7]. Pathologic complete response (pCR), defined as the absence of invasive cancer in both the breast and axillary lymph nodes following neoadjuvant therapy, is recognized as a robust surrogate marker for long-term outcomes, including disease-free survival (DFS) and overall survival (OS) [8]. However, a considerable proportion of patients fail to achieve pCR, emphasizing the need for reliable predictive biomarkers to guide individualized therapy and improve prognostic accuracy [9].

Recent research has increasingly focused on the tumor microenvironment and its immunologic components. Tumor-infiltrating lymphocytes (TILs), representing the host immune response to tumor cells, have emerged as key prognostic and predictive markers in breast cancer [10]. Several studies have demonstrated that higher baseline levels of TILs are associated with better response to neoadjuvant therapy, particularly in triple-negative (TNBC) and HER2-positive subtypes, which are known to be more immunogenic [11-13]. Studies reported that patients with high TIL levels had significantly higher pCR rates compared with those with low TILs [11,12,14].

Importantly, a large pooled analysis of 3,771 patients by Denkert et al. demonstrated that higher concentrations of TILs predicted a greater likelihood of pCR and longer survival in TNBC and HER2-positive breast cancers, while paradoxically serving as an adverse prognostic indicator in luminal-HER2-negative subtypes [15]. This seminal work established the foundation for the current consensus that TILs confer favorable outcomes in immunogenic breast cancer subtypes such as TNBC and HER2-positive disease but not in luminal tumors, likely due to inherent differences in immune microenvironment and tumor antigenicity.

Furthermore, dynamic alterations in TIL levels during neoadjuvant therapy have been correlated with improved pathological response and survival, particularly among TNBC cohorts [16,17]. These findings underscore the dual predictive and prognostic value of TILs, reflecting their role as an immunologic surrogate of treatment sensitivity and host-tumor immune engagement.

Given the molecular heterogeneity of breast cancer and the variable efficacy of systemic therapies, the evaluation of stromal TILs offers a promising biomarker for stratifying patients and guiding treatment optimization. Nevertheless, most existing evidence arises from Western cohorts, and the predictive significance of TILs in ethnically diverse or resource-limited settings remains underexplored. Therefore, the present study was designed to evaluate the association between stromal TIL levels and pathological complete response following neoadjuvant therapy in breast cancer, and to determine whether TILs independently predict pCR across molecular subtypes in a South Asian population.

## Materials and methods

The study was devised as a cross-sectional analytical study at the Department of Oncology, Sindh Medical College, Karachi, Pakistan. The study period was from February 15, 2025, to August 15, 2025. The researcher obtained the necessary ethical approval from the Ethical Research Committee (ERC) of Sindh Medical College, Karachi (Approval No. CM/ERC/2024/48, Date: 11-02-2025).

The sample size was determined using OpenEpi software (Version 3.01). The calculation was based on a meta-analysis by Sun et al. (2024), which reported a pCR rate of 48% in high-TIL tumors versus 27.7% in low-TIL tumors [8]. Using a 95% confidence level, 5% margin of error, and 80% power, a minimum sample size of 102 patients was estimated. To accommodate potential data loss or incomplete records, the sample size was increased to 110 patients.

Participants were selected through non-probability consecutive sampling, including all eligible patients presenting during the study period. Inclusion criteria were: women aged ≥18 years with histologically confirmed breast cancer who received standard neoadjuvant chemotherapy, with or without targeted therapy, and had available pre-treatment biopsy samples. In this study, targeted therapy specifically refers to anti-HER2 agents (trastuzumab alone or in combination with pertuzumab) administered concurrently with chemotherapy in HER2-positive patients. Patients with hormone receptor-positive HER2-negative or triple-negative breast cancer received chemotherapy alone, as targeted therapy or immune checkpoint inhibitors were not part of the standard neoadjuvant regimen during the study period.

Exclusion criteria included incomplete clinical or pathological data, metastatic or recurrent disease at presentation, prior systemic therapy before neoadjuvant treatment, or insufficient tissue for TIL evaluation. Written informed consent was obtained from all participants.

To ensure accuracy of both clinical and histopathological parameters, data collection was performed in two systematic stages. In the first phase, clinical and demographic data were retrieved from hospital medical records, including age at diagnosis, menopausal status, tumor size, histological subtype, tumor grade, clinical stage, hormone receptor (estrogen and progesterone) status, HER2 status, and details of the neoadjuvant regimen. Patient identifiers were coded to maintain confidentiality, and all information was recorded in a structured proforma.

In the second phase, tumor tissue samples from pre-treatment biopsies and post-treatment surgical specimens were re-evaluated for histopathological parameters, including TILs. Formalin-fixed, paraffin-embedded (FFPE) tissue blocks were sectioned at 4 µm and stained with hematoxylin and eosin (H&E). Stromal TILs were assessed following the International Immuno-Oncology Biomarker Working Group (IIOBWG) guidelines [18,19]. Briefly, TILs were defined as the percentage of mononuclear immune cells, including lymphocytes and plasma cells, present within the stromal area of invasive tumor nests, while excluding areas of necrosis, crush artifact, and tertiary lymphoid structures. 

Two board-certified pathologists independently evaluated all slides, blinded to treatment outcomes. Any discrepancy greater than 10% between observers was resolved by joint review, and inter-observer agreement was quantified using Cohen’s kappa statistic (κ = 0.82), indicating strong concordance. Based on a pre-specified cut-off of 20%, TILs were categorized as low (<20%) or high (≥20%), consistent with prior regional validation and international recommendations.

All slides were independently assessed by two board-certified pathologists blinded to treatment outcomes. Discrepancies greater than 10% were resolved through joint review and consensus. Inter-observer agreement was quantified using Cohen’s kappa statistic (κ = 0.82), indicating strong concordance. TILs were categorized into low (<20%) and high (≥20%) groups based on a pre-specified 20% cut-off, which aligns with IIOBWG recommendations and several large-scale studies [12-16,20]. Although some recent studies have explored higher thresholds (e.g., 30%), the 20% criterion was selected to maintain methodological consistency with earlier evidence and enhance comparability across datasets, particularly within South Asian populations. Molecular subtypes (luminal A, luminal B, HER2-enriched, and triple-negative) were classified according to the St. Gallen International Expert Consensus criteria, based on immunohistochemical assessment of ER, PR, HER2, and Ki-67 expression [21].

Following completion of neoadjuvant therapy, surgical specimens were examined for response assessment. pCR was defined as the absence of residual invasive carcinoma in both the breast and axillary nodes, permitting the presence of ductal carcinoma in situ (DCIS) (ypT0/Tis ypN0) [22]. Pathology reports were reviewed to confirm pCR or residual disease status. Clinical and pathological data were cross-verified and entered into a secured database, with double data entry and periodic quality checks to ensure accuracy and completeness.

**Figure 1:**
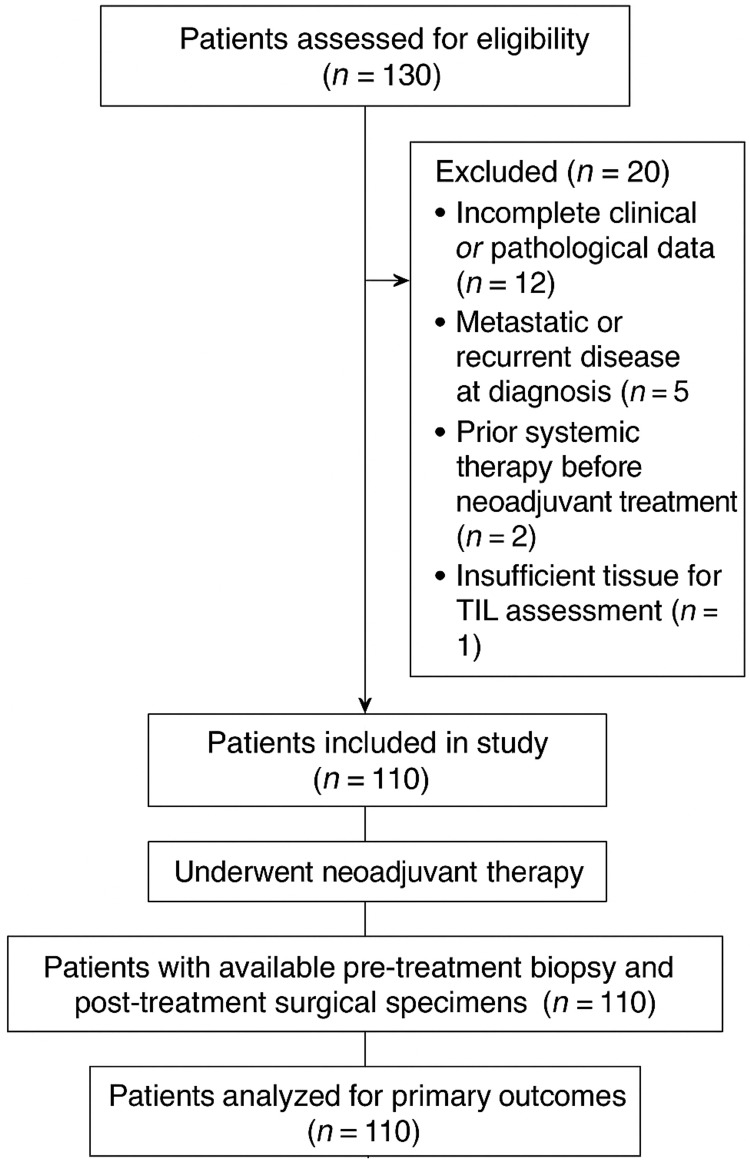
Strengthening the Reporting of Observational Studies in Epidemiology (STROBE)-style flow diagram illustrating the number of patients screened, excluded (with reasons), and included in the final analysis.

Statistical analysis was performed using SPSS version 26.0 (IBM Corp., Armonk, NY, USA). Categorical variables were summarized as frequencies and percentages, while continuous variables were expressed as mean ± standard deviation (SD). The Chi-square test was applied to evaluate the association between pCR and TIL categories (low vs. high). Multivariable logistic regression analysis was conducted to identify independent predictors of pCR while statistically controlling for potential confounding variables, including patient age, molecular subtype, tumor grade, and clinical stage. These variables were selected a priori based on their established influence on treatment response. Adjusted odds ratios (aORs) and 95% confidence intervals (CIs) were calculated to quantify the strength of associations. A two-tailed p-value <0.05 was considered statistically significant. The overall study flow and patient inclusion process are illustrated in Figure 1.

## Results

The mean age of the patients was 48.6 ± 10.2 years. Nearly one-quarter of the patients were younger than 40 years (25.5%), while 30.9% were between 40 and 49 years of age, and 43.6% were aged 50 years or above. Regarding menopausal status, 47.3% of the patients were premenopausal, whereas 52.7% were postmenopausal. Only 20.0% of the individuals reported having a family history of breast cancer, whereas 80.0% did not (Table 1).

**Table 1:** Baseline Demographic and Clinical Characteristics of Patients Diagnosed With Breast Cancer This table summarizes the demographic background of patients included in the study. Age and menopausal status were extracted from hospital medical records at the time of diagnosis. Family history was documented based on self-report during the initial clinical interview, reflecting whether any first-degree relatives had previously been diagnosed with breast cancer.

Variable	Category	n(%)
Age (years)	Mean ± SD	48.6 ± 10.2
Age groups	<40 years	28 (25.5%)
	40–49 years	34 (30.9%)
	≥50 years	48 (43.6%)
Menopausal status	Premenopausal	52 (47.3%)
	Postmenopausal	58 (52.7%)
Family history of breast cancer	Yes	22 (20.0%)
	No	88 (80.0%)

More than half of the patients presented with smaller tumors classified as T1-T2 (58.2%), while 41.8% had larger tumors (T3-T4). The majority of cases were invasive ductal carcinoma (83.6%), followed by invasive lobular carcinoma (9.1%) and other histological subtypes (7.3%). Regarding tumor grade, 10.9% were grade I, 50.9% were grade II, and 38.2% were grade III. Analysis of molecular subtypes showed that luminal A and luminal B accounted for 18.2% and 23.6% of cases, respectively, while HER2-positive and triple-negative breast cancer were observed in 29.1% each. Most patients (70.9%) received chemotherapy alone as neoadjuvant treatment, whereas 29.1% were treated with a combination of chemotherapy and targeted therapy (Table 2).

**Table 2:** Distribution of Tumor Characteristics and Neoadjuvant Treatment Regimens Tumor size and stage were assessed clinically by the attending oncologist through physical examination and breast ultrasound or mammography. Histological type and grade were determined from pretreatment core biopsies by institutional pathologists. Molecular subtyping (Luminal A/B, HER2+, TNBC) was based on local immunohistochemistry reports. Neoadjuvant regimen information was retrieved from oncology treatment records.

Variable	Category	n (%)
Tumor size (clinical stage)	T1–T2	64 (58.2%)
	T3–T4	46 (41.8%)
Histological subtype	Invasive ductal carcinoma	92 (83.6%)
	Invasive lobular carcinoma	10 (9.1%)
	Others	8 (7.3%)
Grade	I	12 (10.9%)
	II	56 (50.9%)
	III	42 (38.2%)
Molecular subtype	Luminal A	20 (18.2%)
	Luminal B	26 (23.6%)
	HER2-positive	32 (29.1%)
	Triple-negative (TNBC)	32 (29.1%)
Neoadjuvant regimen	Chemotherapy only	78 (70.9%)
	Chemotherapy + Targeted Therapy (TT)	32 (29.1%)

When categorized using the 20% cut-off, more than half of the patients demonstrated low levels of tumor-infiltrating lymphocytes (54.5%), whereas 45.5% had high TILs (Table 3).

**Table 3:** Distribution of Tumor-Infiltrating Lymphocytes (TILs) in Pretreatment Biopsies TILs were quantified manually on hematoxylin and eosin (H&E) stained slides by two independent pathologists. The percentage of lymphocytic infiltration in the stromal area adjacent to invasive tumor cells was visually estimated. A threshold of 20% was used to categorize tumors as having “low” or “high” TIL density, based on the consensus method used in our pathology department.

TILs Category (Cut-off 20%)	n (%)	χ²	p-value
Low TILs (<20%)	60 (54.5%)	0.91	0.34
High TILs (≥20%)	50 (45.5%)		

Overall, a pCR was achieved in 36.4% of patients, while 63.6% had residual disease following neoadjuvant therapy. Stratification by TILs showed that patients with high TILs (≥20%) demonstrated a markedly higher pCR rate (56.0%) compared to those with low TILs (<20%) (20.0%). Among patients who did not achieve pCR, a larger proportion of high TIL tumors exhibited partial response (30.0%) compared to low TIL tumors (41.7% of low TIL patients had partial response, lower tumor shrinkage). This indicates that elevated TILs are associated with improved treatment response overall, not just complete response (Table 4). The association between high TILs and pCR was statistically significant (p < 0.001).

**Table 4:** Association Between Tumor-Infiltrating Lymphocytes (TIL) Levels and Pathologic Response After Neoadjuvant Therapy Pathologic complete response (pCR) was defined as the absence of residual invasive tumor in both breast and axillary lymph nodes in surgical specimens following completion of neoadjuvant therapy. Partial response (non-pCR) indicated measurable but incomplete tumor regression. All histopathological assessments were conducted at our institution’s pathology department following standard local reporting guidelines. * p < 0.05 was statistically significant.

Variable	Response	n (%)	χ² (df)	p-value (Chi-square)	Adjusted OR (95% CI)	χ²	p-value
TILs ≥20%	pCR	28 (56.0%)	29.60 (1)	<0.001	5.25 (2.10–13.1)	14.22	<0.001*
	Partial response (non-pCR)	15 (30.0%)	—	—	—	—	—
TILs <20%	pCR	12 (20.0%)	—	—	—	—	—
	Partial response (non-pCR)	25 (41.7%)	—	—	—	—	—
Age <50 years	pCR	15 (40.5%)	2.43 (1)	0.12	1.42 (0.60–3.35)	0.67	0.41
HER2-positive	pCR	12 (50.0%)	4.21 (1)	0.04	2.10 (0.90–4.92)	3.08	0.08
Triple-negative	pCR	8 (38.1%)	1.44 (1)	0.23	1.75 (0.65–4.72)	1.22	0.27

Multivariate logistic regression analysis identified high TILs as an independent predictor of pCR, with patients in this group being nearly five times more likely to achieve a complete pathological response compared to those with low TILs (aOR = 4.75, p < 0.001). In addition, molecular subtype was significantly associated with treatment response; HER2-positive tumors and triple-negative breast cancers showed higher odds of achieving pCR compared to luminal subtypes, with adjusted odds ratios of 2.62 (p = 0.043) and 3.18 (p = 0.012), respectively. Conversely, age and tumor grade did not show statistically significant associations with treatment outcome in the adjusted analysis (Table 5).

**Table 5:** Multivariate Logistic Regression Analysis of Predictors of Pathologic Complete Response (pCR) This table presents multivariate logistic regression results evaluating independent predictors of achieving pCR after neoadjuvant therapy. Variables with p<0.1 in univariate analysis were included in the model. Data on receptor status and tumor-infiltrating lymphocytes (TILs) were obtained from local histopathology reports, and treatment response was verified from surgical pathology findings. Adjusted odds ratios (ORs) represent the relative likelihood of achieving pCR compared to the reference categories. * p < 0.05 is statistically significant. TNBC: triple-negative breast cancer

Predictor		Adjusted OR	95% CI	p-value
Age (<50 vs ≥50 years)		1.45	0.72 – 2.91	0.294
Molecular subtype	HER2-positive vs Luminal	2.62	1.05 – 6.53	0.043
	TNBC vs Luminal	3.18	1.25 – 8.09	0.012
	High TILs (≥20% vs <20%)	4.75	2.03 – 11.09	<0.001*
	Tumor grade (III vs I–II)	1.92	0.88 – 4.20	0.100

## Discussion

In this study, high tumor-infiltrating lymphocytes (TILs ≥ 20%) were strongly associated with pCR to neoadjuvant therapy: patients with high TILs achieved pCR in 56.0% of cases versus 20.0% among those with low TILs, and high TILs remained an independent predictor of pCR in multivariate analysis. These findings are consistent with accumulating evidence that TILs represent a robust immunologic marker of chemosensitivity in breast cancer [23,24].

A sizable meta-analysis by Li et al. [25] found that increased pre-treatment TILs are linked to higher pCR rates across breast cancer subtypes, reinforcing the predictive value observed in our cohort. Clinically, this supports real-world observations that tumors exhibiting stronger immune infiltration often respond more dynamically to systemic therapy [25]. The greater the degree of lymphocytic infiltration, the greater the probability of achieving a complete pathological response, underscoring the biological relevance of an active tumor-immune microenvironment.

In our subgroup analysis, patients who received combined chemotherapy plus targeted therapy (CT + TT), particularly those with HER2-positive disease, demonstrated higher pCR rates (49.0%) compared with those receiving chemotherapy alone (31.5%). A similar pattern was observed for TILs, as tumors with higher TIL levels were more prevalent in the CT + TT subgroup, suggesting a potential interaction between immune activation and HER2-targeted regimens. These trends, while not statistically powered for definitive inference, align with prior studies showing that HER2 blockade may enhance antitumor immune responses and amplify the predictive value of TILs [26].

The immunologic mechanisms underlying these associations likely involve cytotoxic CD8⁺ T-cell-mediated tumor cell killing and modulation of immune checkpoint pathways. Chemotherapy and targeted agents can increase tumor antigen release and reduce immunosuppressive signaling, thereby augmenting cytotoxic T-cell infiltration. Concurrently, immune checkpoints such as programmed cell death protein 1 (PD-1)/programmed death-ligand 1 (PD-L1) and cytotoxic T-lymphocyte-associated protein 4 (CTLA-4) regulate this balance, explaining why tumors with abundant effector T cells or adaptive immune activation exhibit greater chemosensitivity and improved pCR [14,27].

Subtype-specific analyses in recent studies further contextualize our findings. Ciarka et al. [26] summarized evidence showing that the predictive impact of TILs varies by molecular subtype - most pronounced in TNBC and HER2-positive disease, and less consistent in hormone receptor-positive tumors. The higher odds of pCR for TNBC and HER2-positive subtypes in our model mirror this pattern, suggesting that TILs act synergistically with intrinsic tumor biology to modulate therapy response. Conversely, in the TRAIN-2 trial, Liefaard et al. [27] reported no significant association between TILs and pCR in dual-HER2 blockade, likely reflecting ceiling effects of highly efficacious regimens and differences in immune checkpoint modulation.

Recent single-institution and pooled cohort analyses corroborate our principal observation. Rosa et al. [16] and Leon-Ferre et al. [28] both demonstrated that higher baseline or dynamic increases in TILs during therapy predicted superior pCR and survival outcomes in TNBC. Our findings are congruent with these reports, supporting TILs as a practical biomarker of treatment responsiveness.

Methodological consistency and TIL scoring approaches influence comparability between studies. Thomas et al. [29] emphasized that reproducible TIL scoring enhances the prognostic precision of residual cancer burden assessments. In practice, closer collaboration between oncologists and pathologists is essential to ensure standardized interpretation and consistent clinical application of TIL scoring. Comprehensive reviews and pooled analyses, including that of Alamoodi et al. [30], confirm the reproducible association between high TILs and improved pCR, though they urge prospective standardization before routine clinical adoption.

The strong and independent association between high TILs and pCR highlights the potential of TIL assessment as a practical, low-cost biomarker in breast cancer management. Routine pre-treatment evaluation of TILs using standardized hematoxylin-and-eosin-based scoring could help identify patients more likely to benefit from neoadjuvant therapy, particularly within TNBC and HER2-positive subsets. In low- and middle-income countries, where genomic and molecular assays are often unavailable, TIL assessment represents a cost-effective and widely accessible tool for guiding treatment decisions. Integrating TIL scoring into standard pathology workflows can inform resource allocation, prioritize candidates for more intensive regimens, and reduce overtreatment, thus enhancing both clinical outcomes and system efficiency in resource-limited settings.

A major strength of this study is the control of key clinical and pathological confounding variables, including patient age, tumor grade, molecular subtype, and clinical stage, through multivariable analysis. This statistical adjustment strengthens the reliability of the observed associations between TIL levels and treatment response. However, as a retrospective study, it remains susceptible to potential residual confounding from unmeasured factors such as Ki-67 index, type and duration of hormone therapy, and the number of chemotherapy cycles administered. Therefore, while the findings provide robust evidence of independent associations, causal relationships cannot be definitively inferred, and future prospective studies are recommended to validate these results.

## Conclusions

Higher stromal TILs were strongly associated with improved pCR in patients receiving neoadjuvant therapy for breast cancer, with the predictive effect being most pronounced in HER2-positive and triple-negative subtypes. These results reinforce the prognostic and predictive value of immune infiltration and align with global evidence supporting TILs as a reliable biomarker of chemosensitivity.

Our findings advocate for the integration of TIL assessment into routine pathology reports to enhance prognostic evaluation and inform therapeutic planning. By validating TILs as a predictive biomarker in a South Asian cohort, this study contributes meaningful regional data to the international oncology literature and highlights its practical utility in guiding personalized treatment decisions. Future prospective and translational studies should explore the synergistic potential of combining TIL quantification with molecular profiling and immune-based therapeutic strategies to further optimize individualized care and improve clinical outcomes.
